# Alteration of NMDA receptor trafficking as a cellular hallmark of psychosis

**DOI:** 10.1038/s41398-021-01549-7

**Published:** 2021-08-30

**Authors:** Agnès Espana, Henrik Seth, Julie Jézéquel, Tingting Huang, Delphine Bouchet, Marylin Lepleux, Hélène Gréa, Karl Bechter, Marion Schneider, Eric Hanse, Laurent Groc

**Affiliations:** 1 Université de Bordeaux, CNRS, Interdisciplinary Institute for Neuroscience, UMR 5297, F-33000 Bordeaux, France; 2 Department of Physiology, Institute of Neuroscience and Physiology, The Sahlgrenska Academy, University of Goteborg, Gothenburg, Sweden; 3 Clinic for Psychiatry and Psychotherapy II, Ulm University, Bezirkskrankenhaus Günzburg Ludwig-Heilmeyer-Str. 4, D-89312 Günzburg, Germany; 4 Division of Experimental Anesthesiology, University Hospital Ulm, 89081 Ulm, Germany

**Keywords:** Molecular neuroscience, Schizophrenia

## Abstract

A dysfunction of the glutamatergic transmission, especially of the NMDA receptor (NMDAR), constitutes one of the main biological substrate of psychotic disorders, such as schizophrenia. The NMDAR signaling hypofunction, through genetic and/or environmental insults, would cause a neurodevelopmental myriad of molecular, cellular, and network alterations that persist throughout life. Yet, the mechanisms underpinning NMDAR dysfunctions remain elusive. Here, we compared the membrane trafficking of NMDAR in three gold-standard models of schizophrenia, i.e., patient’s cerebrospinal fluids, genetic manipulations of susceptibility genes, and prenatal developmental alterations. Using a combination of single nanoparticle tracking, electrophysiological, biochemical, and behavioral approaches in rodents, we identified that the NMDAR trafficking in hippocampal neurons was consistently altered in all these different models. Artificial manipulations of the NMDAR surface dynamics with competing ligands or antibody-induced receptor cross-link in the developing rat brain were sufficient to regulate the adult acoustic startle reflex and compensate for an early pathological challenge. Collectively, we show that the NMDAR trafficking is markedly altered in all clinically relevant models of psychosis, opening new avenues of therapeutical strategies.

## Introduction

Psychotic disorders, such as schizophrenic spectrum disorders (SCZSD) [1], are life-threatening mental illnesses that impose a substantial burden of morbidity and mortality. Epidemiological and biological studies support the hypothesis that a dysfunctional interplay between genetic and environmental factors profoundly perturbs synaptic and brain network functions [2, 3]. These dysfunctions likely originate during childhood as the development of glutamatergic, GABAergic, and monoaminergic circuits are particularly sensitive to genetic and environmental alterations [4, 5]. The glutamatergic hypothesis of SCZSD has received a lot of attention and is supported by a wealth of reports. First, glutamatergic N-methyl-D-aspartate receptor (NMDAR) gene expression and protein levels are altered in post-mortem brain tissue from SCZSD patients [6–9]. Second, NMDAR antagonists mimic psychotic-like behaviors in healthy humans and rodents [6–9]. Third, genetic ablation of NMDAR in developing pyramidal or inhibitory neurons induces psychotic-like behavioral deficits at adult stage in rodents [10–12]. Mutations of NMDAR subunits have also been described in few cases of patients with psychosis [13], although genome-wide association studies mainly highlight alterations of proteins associated with synaptic (e.g., signaling cascades), metabolic, and immune functions [14, 15]. Yet, due to the complexity of psychotic disorders’ etiology, the molecular and cellular mechanisms underpinning NMDAR dysfunction remain unclear.

NMDAR are heterotetramers consisting of the association between the obligatory GluN1 subunit with GluN2A-D and/or GluN3 subunits, providing specific biophysical and pharmacological properties to each receptor subtype [16]. Throughout development, the synaptic NMDAR composition changes from GluN2B subunit to a predominance of GluN2A subunit. This activity-dependent change in NMDAR subtypes affects the plasticity of developing synapses and plays a central role in the fine-tuning of neuronal network formation [16, 17]. NMDAR are constantly exocytosed and endocytosed to/from the plasma membrane in which they laterally diffuse and highly exchange between sub-compartments, such as between the postsynaptic and extrasynaptic areas [18, 19]. Besides modulating the NMDAR synaptic pool, this fast dynamics also plays an instrumental role in the establishment of synaptic long-term potentiation at maturing synapses and during associative memory formation [20–23]. Recently, the presence of autoantibodies directed against NMDAR (NMDAR-Ab) in patients with anti-NMDAR encephalitis and SCZSD was shown to alter NMDAR surface dynamics, disrupting NMDAR-dependent synaptic plasticity and associative memory [24–28], although the prevalence of these autoantibodies in patients with first episode psychosis and the relation between antibody findings and psychiatric disorders remain to be broadly established [29]. The causality between antibody findings and psychotic disorders does not least depend from the broadness of parameter panel and quality of cerebrospinal fluid (CSF) analysis [30]. Yet, the NMDAR-Ab provides additional evidence that a specific alteration of NMDAR signaling in humans is involved in behavioral alterations related to psychosis [31].

A deficit in NMDAR synaptic signaling can thus have multiple origins, from genetic mutation of the channel to altered trafficking to/within synapses. In other words, an altered NMDAR signaling could be a common marker of psychosis. Over the past four decades, strong effort has been made to produce valid and clinically relevant animal models of psychosis. These have been critical to investigate the basis of neuropsychiatric disorders since they allow the identification of molecular and cellular aspects of the pathophysiology. Besides the use of human brain material, three main “classes” of models have emerged from these translational attempts: (1) pharmacological manipulation of main neurotransmitter systems, (2) genetic manipulations of human susceptibility genes (e.g., Disrupted-in-Schizophrenia 1 (DISC1) or neuregulin mutations), and (3) developmental manipulations (e.g., neuronal activity blockade, infection, trauma) in which synaptic and network assembly are corrupted during a critical neonatal period [32–36]. In this study, we identified NMDAR trafficking alterations as a convergence point between several gold-standard models of psychosis.

## Material and methods

### Patients’ cerebrospinal fluid (CSF)

CSF was obtained from patients with SCZSD (*N* = 12), and affective spectrum disorders (AffectSD; *n* = 9), subarachnoid hemorrhage (SAH; *n* = 9 patients), brain polytrauma (*n* = 3), hemophagocytic lymphohistiocytosis (*n* = 1) patients were collected at symptom presentation. The CSF of patients with SAH were collected at the time of almost complete resolution and inflammation. Cell-free CSF aliquots were and stored at −80 °C. For patients suffering from SCZSD or AffectSD, the study was approved by the Ethics Committee of Ulm University (No. 41/2001) as previously published [37], SAH patients samples were collected within a another study, also approved by the Ethics Committee of Ulm University (ID: 021-07122010). Psychiatric patients were included when hospitalized. Recruitment took place during a period of 5 years at the Department Psychiatry and Psychotherapy II, Günzburg, Ulm University. Inclusion criteria were SCZSD or AffectSD (ICD-10 F20-F25 and F30-F33), aged 18–65 years, and informed written consent. All patients received multiple treatments without major substantial long-term improvement. Exclusion criteria: increased bleeding risk, increased cerebral pressure (excluded by brain imaging), fever, leukopenia, suspected meningoencephalitis, or multiple sclerosis.

### Animals and injections

Animal procedures were conducted in accordance with the European Community guidelines (Directive 2010/63/EU) regulating animal research, and were approved by the local Bordeaux Ethics Committee or local ethics committee of Gothenburg (permit: 167-2015). All animals were raised in our animal facility in compliance with ethical guidelines. Pregnant female rats were purchased from Janvier (France) or obtained from our in-house breeding (Gothenburg). Embryonic day 17 (E17) Sprague-Dawley or Wistar pregnant rats were injected intraperitoneally with 26 mg kg^−1^ of methylazoxymethanol acetate (MAM) (MRI, Missouri) or saline vehicle. To assess the number of cell progenitor, 50 mg kg-1 of BrDU was also intraperitoneally injected concomitantly (Roche, Switzerland). For mice experiments, female *il1rapl1*−/*y* and their control +/*y* littermates (C57BL/6 background), housed in 12/12 LD with ad libitum feeding, were used. Every effort was made to minimize the number of animals used and their suffering.

### Neuronal cultures, transfection, and treatments

Mixed cultures of hippocampal neurons and glia cells were prepared from embryonic day 18 (E18) or postnatal day 2 (P2) rat or mice pups, as previously described [38]. Briefly, hippocampi were dissected and mechanically dissociated after 15 min treatment at 37 °C with 0.05% trypsin-EDTA 1X (Fisher, France). Cells were plated at a density of 300,000 cells per dish onto poly-l-lysine pre-coated glass coverslips (1 mg ml^−1^, Sigma, France). Medium consisted in MEM 1X with Earle’s salt (Invitrogen, France) supplemented with 10% NU serum (BD Biosciences, France), 0.8% glucose (Sigma), 1 mM sodium pyruvate (Sigma), 2 mM glutamine (PAA, France), Neuromix 1X (PAA, France) and 10 IU ml^−1^ penicillin-streptomycin (Fisher). At day in vitro 3 (D3), half medium was replaced and 1.7 µM of Ara-C (Sigma, France) was added in the dishes. Cultures were kept at 37 °C in 5% CO_2_. Neurons were transfected using Effectene (Qiagen, Hilden, Germany) according to the manufacturer’s recommendations. Neurons were transfected at D7-10, with Homer 1c-dsred, Homer 1c-GFP, GluN2A-SEP, GluN3A-SEP, GluA1-SEP, GABA_A_-GFP, or Dopamine D1-YFP (1 µg cDNA/dish). For Disc-1 expression or downregulation, cell cultures were transfected at D7-10 with GluN2B-SEP (0.5 µg cDNA/dish), two Disc-1 RNAis (0.75 µg/dish for each RNAi), or Scramble RNAi (Scr RNAi, 1.5 µg/dish). Single nanoparticle tracking was performed 36 h after transfection (D8–12) or one week (D14-16) after transfection.

### Immunocytochemistry

Endogenous surface GluN2B-NMDAR or transfected GluN2A-SEP, GluN3A-SEP, GluA1-SEP, GABA-gamma2-GFP, or Dopamine D1-YFP were immune stained in live neurons using a rabbit polyclonal anti-GluN2B (1:200; Alomone, Israel) or mouse monoclonal anti-GFP (1:500, Roche, Switzerland), respectively. Live surface staining (10 min at 4 °C) was followed by 15 min fixation in 4% paraformaldehyde (PFA), quenching in 50 mM NH4Cl for 10 min, blocking for 1 h, and incubation with secondary Ab’s coupled to Alexa fluorophores for 1 h in 1% bovine serum albumin (BSA) (Sigma-Aldrich, Missouri, USA) at room temperature. Neurons were incubated with anti-rabbit (1:500, 30 min, Invitrogen) or anti-mouse (1:500, 30 min, Invitrogen) alexa 488 antibodies, respectively. Cells were mounted in Mowiol (Calbiochem, Merck) or Vectashield^®^ (Vector Laboratories, Burlingame, CA). For intracellular labeling, cells were fixed with PFA 4%, permeabilised with 0.1% Triton (TritonX-100, Sigma), and incubated (1–2 h) with polyclonal rabbit anti-GFAP (1:1000, Dako, France), monoclonal mouse anti-NeuN (1:1000, Millipore), anti-GAD67 (1:500, Sigma), or polyclonal guinea pig anti-Homer (1:500, Synaptic Systems, Germany). Then, cells were incubated with secondary antibodies (30 min, 1:500): anti-rabbit alexa 568, anti-mouse alexa 488 or 568 (Invitrogen), or anti-guinea-pig alexa 594 (Jackson Immunoresearch). Between incubations, coverslips were washed three times in PBS. Coverslips were mounted in Vectashield (Vector Laboratories, Burlingame, CA). For immunohistochemistry in brain slices, P2 pups were intracardiacally perfused with 4% PFA and removed. After rinses in PBS, brains were kept in 30% sucrose solution overnight, frozen at −55 °C and sliced with a cryostat (20 µm thick; Leica, France). For BrDU immunostaining, slices were first heated at 37 °C (30 min) and incubated in HCl (1 N, 7 min, 37 °C) before incubation with mouse anti-BrDU antibody (1:500, DSHB, Iowa) followed by an anti-mouse alexa 488 (1:500, Invitrogen). All coverslips and slices were mounted in Mowiol. For cells counting, slices from control and MAM pups were selected in similar hippocampal area. Finally, immunofluorescence images were randomly selected for analysis and collected on a video confocal spinning-disk system (Leica DMI6000B, Wetzlar, Germany) with a CoolSNAP HQ2 camera (Photometrics, Tucson, USA). For surface cluster analysis, dendritic branches were chosen manually in a blinded manner and cluster areas and numbers were obtained using a manual threshold approach. Immunofluorescence intensity and linear density were calculated using ImageJ (NIH). For all type of experiments, a minimum of three independent cultures was used per condition.

### Single nanoparticle tracking

Single nanoparticle (Quantum Dot, QD; Invitrogen) tracking experiments were performed as previously described [25]. Schematically, neurons were incubated with either rabbit polyclonal anti-GluN2B (Alomone Laboratories; 1:200) or mouse monoclonal anti-GFP (1:500, Roche, Switzerland), followed by an incubation with QD655 coupled to goat anti-rabbit F(ab’)2 (1:10000, Invitrogen). All incubations were made at 37 °C during 10 min in culture medium. Non-specific binding was blocked by adding 1% albumin from BSA (Sigma) during QDs incubation. Synapses were labeled with Mitotracker green (1:10000, 37 °C, 15 s) (Invitrogen). Single QDs and Mitotracker were imaged using a mercury lamp and appropriate excitation/emission filters on a Nikon Eclipse Ti microscope with 100X objective. Signals were detected using an electron-multiplying charge-coupled device camera (Evolve, Photometric, Tuscon, USA). Images were acquired with an integration time of 50 ms. QDs were recorded for at least 500 consecutive frames on randomly selected dendritic regions. Acquisition was made with MetaMorph software (v.7.7.11.0, Molecular Devices, Sunnyvale, USA). The instantaneous diffusion coefficient (D) was calculated for each trajectory from linear fits of the first four points of the mean square displacement (MSD) vs. time function using MSD(t) ≤ r2 > (t) = 4Dt. To determine the distribution of single QD complexes, frame stacks were obtained and after binarisation of the synaptic signal was defined and the QD complexes were automatically located into synaptic or extrasynaptic compartment.

### Electrophysiology

#### Preparation of acute brain slices

P8-15 Wistar rats (controls or from MAM-treated dam, above) were anesthetized with isoflurane and parasagittal brain slices (350 µm) were prepared in an ice-cold sucrose buffer solution containing (in mM): 250 sucrose, 2 KCl, 7 MgCl2, 0.5 CaCl2, 1.15 NaH2PO4, 11 glucose, and 26 NaHCO3 (gassed with 95% O_2_/5% CO_2_). Slices were then incubated for at least 1 h at 33 °C and stored at room temperature in an artificial CSF (ACSF) solution containing (in mM): 124 NaCl, 3 KCl, 2 CaCl_2_, 4 MgCl_2_, 26 NaHCO_3_, 1.25 NaH_2_PO_4_, 0.5 ascorbic acid, 3 myo-inositol, 4 D, L-lactic acid, and 10 D-glucose (gassed with 95% O_2_/5% CO_2_; pH 7.35).

#### Extracellular field EPSP recordings

During recordings, the slice was placed in a submersion-recording chamber and perfused with ACSF at a constant flow (~2 ml m^−1^) at room temperature. The perfusion ACSF contained (in mM) 124 NaCl, 3 KCl, 4 CaCl_2_, 4 MgCl_2_, 26 NaHCO_3_, 1.25 NaH_2_PO_4_, and 10 D-glucose (gassed with 95% O_2_/5% CO_2_; pH 7.35). Picrotoxin (100 µM) was always present in the perfusion ACSF to block GABA_A_ receptors. Electrical stimulation of Schaffer collateral afferents was applied at 0.1 Hz in the stratum radiatum. Two alternating inputs (control/LTP). Stimulation impulses consisted of 200 µs constant current pulses generated by a stimulator (Model DS3, Digitimer Ltd, Letchworth Garden City, UK) and delivered through an insulated tungsten microelectrode (resistance ≈ 0.5 MΩ). Stimulation intensity was set such that there were no signs of action potential activity on the field EPSP (fEPSP). LTP induction consisted of five trains, 20 impulses at 100 Hz. fEPSP recordings were made by means of a glass micropipette filled with 1 M NaCl (resistance ~ 2 MΩ) in the stratum radiatum. fEPSPs were recorded at a sampling frequency of 10 kHz and filtered at 1 kHz, using an Axoclamp 700B amplifier, digitized using a Digidata 1440A (Axon, Molecular Devices, Sunnyvale CA, US) and binned in groups of three synaptic responses.

#### Whole-cell patch-clamp recordings

Whole-cell patch-clamp recordings were performed on CA1 pyramidal cells as previously described [39]. The pipette solution contained (in mM): 130 Cs-methanesulfonate, 2 NaCl, 20 HEPES, 0.6 EGTA, 5 QX-314, 4 Mg-ATP, and 0.4 GTP (pH ~7.3 and osmolality 280–300 mOsm). During recordings, the slice was placed in a submersion-recording chamber and perfused with ACSF at a constant flow (~2 ml m^−1^) at room temperature. The perfusion ACSF contained (in mM) 124 NaCl, 3 KCl, 2 CaCl_2_, 1 MgCl_2_, 26 NaHCO_3_, 1.25 NaH_2_PO_4_, and 10 D-glucose (gassed with 95% O_2_/5% CO_2_; pH 7.35). Picrotoxin (100 µM) was added to inhibit GABA_A_ receptor channels. Patch pipette resistances were 3–5 MΩ. Current was recorded at a sampling frequency of 10 kHz and analog filtered at 3 kHz, using an EPC-10 amplifier (HEKA Elektronik, Lambrecht, Germany). The liquid junction potential was both measured and calculated to be about 8 mV and was not corrected for. Series resistance was monitored using a 20 ms 10 mV hyperpolarizing pulse. The series resistance was not allowed to exceed 20 MΩ in whole-cell recordings, or to change more than 20% during an experiment, otherwise the experiment was discarded. For recording of spontaneous AMPAR (−70 mV) and NMDAR (+40 mV) mediated responses, receptor kinetics as well as tonic APV-sensitive currents, we patched pyramidal cells within the CA1 cell layer. Tonic NMDA currents were recorded as the subtracted holding current at a depolarized potential of +40 mV before and after the addition of APV. For recording of evoked EPSCs, Schaffer collateral/commissural afferents were stimulated using 0.2 ms biphasic (negative/positive) constant current pulses (5–60 µA; STG 1002, Multi Channel Systems, Reutlingen, Germany) delivered through an insulated tungsten microelectrode (resistance ~ 0.5 MΩ). Stimulation electrodes were positioned in the *stratum radiatum*, at least 100 µm from the recorded cell, and synaptic inputs received test pulse stimulation every 5 s. Evoked and spontaneous responses were analyzed off-line using custom-made IGOR Pro (WaveMetrics, Lake Oswego, OR, USA) software. Receptor kinetics (Tau decay) was analyzed using a single exponential offset curve fit within IGOR Pro. Data are expressed as means ± SEM. Statistical significance for independent samples was evaluated using paired *t*-test or Student’s *t-*test, unless otherwise indicated. Multiple comparisons were corrected for using the modified Holm-Bonferroni algorithm.

### Synaptosomes preparation

Hippocampi from treated rats were homogenized with Teflon glass potter in 3 ml of ice-cold TPS buffer (0.32 M sucrose, 4 mM Hepes pH 7.4 and a protease inhibitor cocktail -1:1000, Calbiochem). After centrifugation at 1000 g for 8 min at 4 °C, the pellet (P1) was saved and the supernatant (S1) centrifuged once again at 12,500 *g* for 13 min at 4 °C. The resulting P2 pellet (crude synaptosomes) was resuspended with 1 ml of TPS buffer and layered on top of two-step sucrose density gradient (0.8 M sucrose in 4 mM Hepes pH 7.4 buffer on top/1.2 M sucrose in 4 mM Hepes pH 7.4 buffer on the bottom). After centrifugation at 50,000 g, 4 °C for 70 min, synaptosomes were located at the interface of the two sucrose solutions, collected, and quantified.

### Western blot analysis

Synaptosomes samples were prepared with 2x sample buffer to load 2 mg of total protein per well. Before loading on a gel, the samples were boiled at 95 °C for 5 min. Samples were separated by SDS/PAGE (4–20% Mini-PROTEAN^®^ TGX Stain-Free™ Gel-Biorad) for 40 min at 200 V. To quantify total protein amount in each lane, and normalized the NMDAR detection between conditions, we activated gels with UV using the Stain-Free technology, and acquired the resulting signal. Gels were then blotted onto nitrocellulose membrane during 1 h at 100 V. After blocking 1 h in 5% milk -Tris-saline-0.05% tween 20 (TBST), the membranes were blotted with an anti GluN2A Ab (1 mg/ml home-made antibody, Agrobio), an anti- GluN2B Ab (1 mg/ml home-made antibody, Agrobio), or an anti-GluN1 Ab (0.25 mg/ml, BDBiosciences), diluted in TBST 0.5% milk, during 1 h at room temperature. Corresponding secondary antibodies were used at 1:10 000 in TBST 0.5% milk. Detection was performed using the SuperSignal West Femto Maximum Sensitivity Substrate detection System (Pierce) revealed with the Chemidoc system (Biorad). Quantification of band intensity was performed using Image Lab software (Biorad), and NMDAR detection was normalized on total protein detection with Stain-Free technology.

### Stereotaxic injection

Stereotaxic injections and animal procedures were conducted in accordance with the European Community guidelines (Directive 2010/63/EU) regulating animal research, and were approved by the local Bordeaux Ethics Committee. P9 or P12 male Sprague-Dawley rats from MAM or control groups were anesthetized by isoflurane inhalation and placed in a stereotaxic frame. For x-link experiments, the control group received 1 µl of anti-rabbit alexa 568 (control IgG, 1/5), while the GluN2B-x-link group received 1 mg of polyclonal rabbit anti-GluN2B subunit (Alomone Labs). For the competing ligand experiments, 1 µl of TAT-NS or TAT-2B peptide (10 µM) were injected. The antibody or TAT-peptides were stereotaxically injected in each hippocampus (coordinates relative to bregma, AP: −3 mm, ML: 2 mm, DV: −2.8 mm).

### Prepulse inhibition (PPI)

Male rats from all groups were tested between P60 and P70. PPI was performed using a Panlab startle chamber (Harvard, San Diego Instruments). The startle chamber contains a transparent Plexiglas to restrain rats. A background noise of 65 dB was maintained throughout session. Each PPI session lasted for ~30 min and began with a 5 min acclimatization period with a constant background noise and 10 startle pulses (120 dB, 40 ms) with 30 s inter-trial interval. The session was composed of 40 randomly presented trials with 30 s inter-trial interval: 10 trials with no stimulus, 10 trials with pre-pulse alone (80 dB, 20 ms duration), 10 trials with startle pulse alone and 10 trials with pre-pulse followed 80 ms later by a startle pulse. Experiments were analyzed with Packwin 2.0 and PPI was defined as the percent reduction in startle amplitude in the prepulse/pulse trial compared to the pulse alone trial and was calculated by (100 × ((S-PP)/S)), where S = average response on startle-only trials and PP = average response on prepulse + startle trials.

### Data and statistical analysis

All statistics were performed with GraphPad Prism 5.03. Statistical tests, sample sizes and *P* values are stated for each experiment in the figure legends or in the results. All data except diffusion coefficient corresponds to normally distributed values. Parametric and non-parametric tests were performed according to the values’ distribution and are indicated in figure legends.

## Results

### Cerebrospinal fluid (CSF) from patients with SCZSD alter NMDAR surface dynamics and organization

**Fig. 1:**
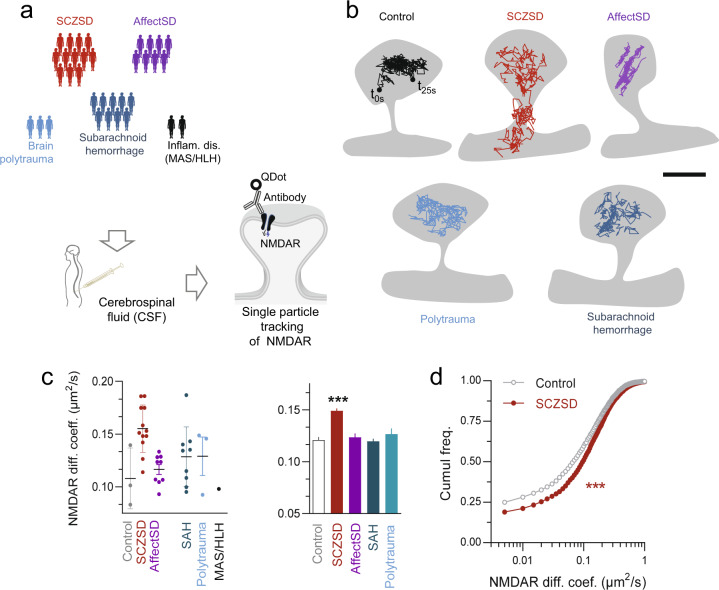
Cerebrospinal fluid (CSF) from patients with SCZSD alters the surface dynamics of synaptic NMDAR. **a** Schematic description of the experimental workflow. CSF from patients with schizophrenic spectrum disorders (SCZSD, *n* = 12), affective spectrum disorders (AffectSD, *n* = 9) subarachnoid hemorrhage (SAH, *n* = 9 patients), brain polytrauma (*n* = 3), hemophagocytic lymphohistiocytosis (HLH, *n* = 1), were collected. Then, single nanoparticle (QDot) tracking of GluN2B-NMDAR was performed onto cultured hippocampal neurons exposed to CSF. **b** Representative trajectories (50 ms acquisition) of surface GluN2B-NMDAR-QD complexes in dendritic spines of neurons exposed to various CSFs. Scale bar = 300 nm. **c** Diffusion coefficient (µm^2^/s) of synaptic GluN2B-NMDAR values after exposure for 15–30 min to patients’ CSF. Each plotted dot corresponds to the median diffusion coefficient value of one patient CSF. The mean and standard deviation (SD) are represented for each condition. *Right panel*, Comparison of GluN2B-NMDAR synaptic diffusion coefficient values after exposure for 15–30 min to patients’ CSF (Control, *n* = 2757 trajectories; SCZSD, *n* = 5849; AffectSD, *n* = 2410; Polytrauma, *n* = 825; SAB, *n* = 3957; ***p < 0.0001, Kruskal–Wallis followed by Dunn’s multiple comparison test). Data are represented as mean ± sem. **d** Cumulative distributions of GluN2B-NMDAR instantaneous diffusion coefficient (µm^2^/s) in control condition and SCZSD CSF (*p* < 0.0001, Kolmogorov–Smirnov test).

**Fig. 2:**
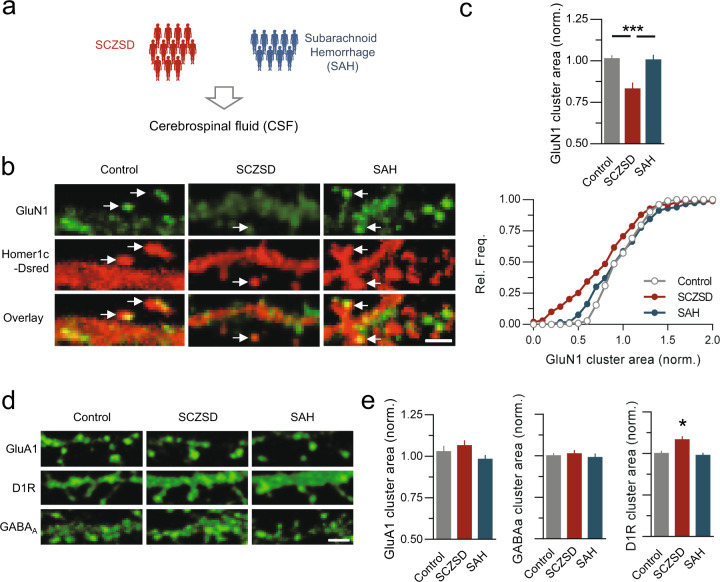
CSF from patients with SCZSD specifically alters NMDAR surface clusters. **a** Schematic description of the experimental workflow, with the collection of CSF from patients with subarachnoid hemorrhage (SAH, *n* = 9 patients) and schizophrenic spectrum disorders (SCZSD, *n* = 12). **b** Surface immunostaining for GluN1-SEP NMDAR (green) onto neurons transfected with Homer1c-DsRed (red) and exposed to SCZSD or SAH CSF. Arrows indicate postsynaptic areas. Scale bar = 1 µm. **c** The area of GluN1-NMDAR surface clusters were significantly reduced by SCZSD CSF (Control, *n* = 103 clusters; SCZSD, *n* = 99; SAH, *n* = 116; *N* = 6–8 neurons per condition; ***p < 0.001, ANOVA 1 followed by Newman–Keuls multiple comparisons test). *Below*, Cumulative distributions of GluN1-NMDAR cluster area between conditions. **d** Surface immunostaining of GluA1-AMPA, Dopamine D1 (D1R), or GABAa receptor onto neurons exposed to SCZSD or SAH CSF. Scale bar = 1 µm. **e** Comparison of the area of surface clusters for GluA1-AMPA receptor (Control, *n* = 129 clusters; SCZSD, *n* = 130; SAH, *n* = 139; *N* = 6–8 neurons per condition), GABAa receptor (Control, *n* = 140; SCZSD, *n* = 182; SAH, *n* = 146; *N* = 6–8 neurons per condition), D1R (Control, *n* = 87; SCZSD, *n* = 100; SAH, *n* = 90; *N* = 6–8 neurons per condition; *p < 0.05, ANOVA 1 followed by Newman–Keuls multiple comparisons test).

In order to test the hypothesis that the CSF of patients suffering from SCZSD can alter the organization and function of NMDAR located at the plasma membrane of neurons, we performed a single nanoparticle tracking experiment in cultured hippocampal neurons. Cells were acutely exposed to CSF collected from patients with various neurological, inflammatory, and psychiatric conditions (Fig. 1a), and the membrane dynamics of synaptic NMDAR were recorded (see “Methods” section). In control condition (untreated neurons), surface NMDAR diffuse along dendritic segments and were more confined in synapses and spines (Fig. 1b), as previously reported [40, 41]. NMDAR surface diffusion was not significantly affected in presence of any of the patients’ CSF but the SCZSD CSF. Indeed, SCZSD CSF induced a destabilization of synaptic NMDAR as witnessed by a 30% increase of NMDAR instantaneous diffusion coefficient (Fig. 1c) and a reduced fraction of immobile NMDAR at the synapse (Fig. 1d). Out of the 12 patients with SCZSD, 10 exhibited this clear upregulation of the NMDAR surface dynamics (two patients exhibited values similar to those in control CSF). Thus, the CSF of patients suffering from SCZSD, but not of major neurological and inflammatory conditions, rapidly and potently displaced surface NMDAR. Such a rapid change in surface dynamics would likely alter the synaptic NMDAR content. To address this possibility, we labeled surface NMDAR in live hippocampal neurons exposed to CSF from either psychiatric (i.e., SCZSD) or subarachnoid hemorrhage condition. As previously shown [38, 40], most of the surface NMDAR clusters colocalise with postsynaptic markers, such as Homer 1c (Fig. 2b). In the presence of SCZSD CSF, the NMDAR cluster area was significantly reduced (Fig. 2c). The cumulative distributions of NMDAR cluster area indicate that the observed decrease with SCZSD CSF is due to a left shift of the whole population (Fig. 2c). In order to test whether the CSF effect was specific for NMDAR, we measured the membrane cluster area of glutamatergic AMPA, dopaminergic D1, and GABAergic GABA_A_ receptors. The membrane and synaptic clusters of AMPA and GABA_A_ receptors remained unaffected by the presence of SCZSD CSF (Fig. 2d, e; Supplementary Fig. 1). It can be noted that dopaminergic D1 receptor cluster slightly increased in the presence of SCZSD CSF (Fig. 2d, e). Collectively, these data show that the CSF of patients suffering from SCZSD—but not of AffectSD, neurological or inflammatory conditions—impair the dynamic organization of synaptic NMDAR in hippocampal neurons, suggesting that NMDAR hypofunction in SCZSD can rely, in part, on a corrupted membrane dynamics of the receptor.

### DISC1 genetic alteration is sufficient to alter NMDAR surface dynamics

**Fig. 3:**
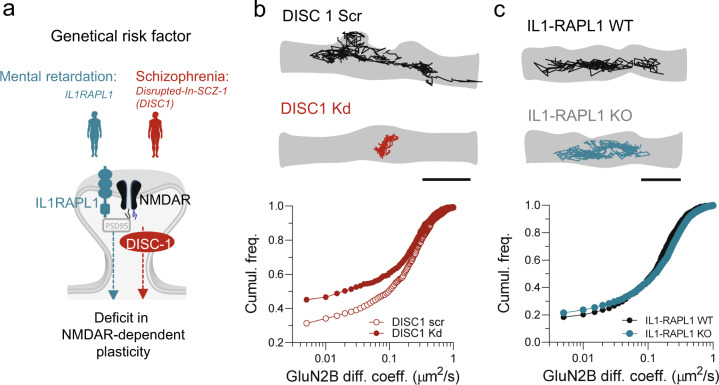
DISC1 downregulation alters NMDAR surface dynamics. **a** Schematic description of the experimental workflow: we compared the GluN2B-NMDAR surface dynamics onto neurons in which we downregulated either IL1RAPL1 or DISC1. **b** Representative trajectories (50 ms acquisition) of surface GluN2B-NMDAR-QD complexes on neurons transfected with either a scrambled siRNA (DISC1 Scr) or DISC1 siRNA (DISC1 Kd). Scale bar = 300 nm. *Below*, Cumulative distributions of synaptic GluN2B-NMDAR instantaneous diffusion coefficient (µm^2^/s) in DISC1 Scr and DISC1 Kd (Scr, *n* = 976 trajectories; Kd, *n* = 662; ***p < 0.0001, Kruskal–Wallis followed by Dunn’s multiple comparison test). **c** Representative trajectories (50 ms acquisition) of surface GluN2B-NMDAR-QD complexes on neurons from IIL1RAPL1 wild-type or KO mice. Scale bar = 300 nm. *Below*, Cumulative distributions of synaptic GluN2B-NMDAR instantaneous diffusion coefficient (µm^2^/s) in WT and KO conditions (WT, *n* = 1430 trajectories; KO, *n* = 1097; *p* > 0.05).

The DISC1 is a robust susceptibility gene for psychiatric disorders, including SCZSD [42, 43]. Mutation of DISC1 impairs NMDAR synaptic transmission and plasticity processes [44–46]. DISC1 is a protein involved in several key functions of the synapse and serves as a multi-scaffold for numerous synaptic proteins [43]. To confront the above CSF results to a gold-standard genetic model of SCZSD, we knocked down the expression of DISC1 in hippocampal neurons using a shRNA approach [47] and investigated its effect on the surface dynamics of synaptic NMDAR. The downregulation of DISC1 expression significantly reduced the surface dynamics of NMDAR, impacting both the fraction of mobile receptors and their diffusion coefficient (Fig. 3b, Supplementary Fig. 2). In contrast, IL1RAPL1 knockout, a genetic model of intellectual disability in which synaptic plasticity is also impaired [48] did not alter NMDAR surface dynamics in hippocampal neurons (Fig. 3c). Thus, a deficit in NMDAR membrane dynamics appears specific to DISC1 model further strengthening the possibility that an alteration of NMDAR dynamic organization is a common marker of SCZSD models.

### NMDAR surface dynamics and synaptic plasticity are developmentally impaired in the MAM model

**Fig. 4:**
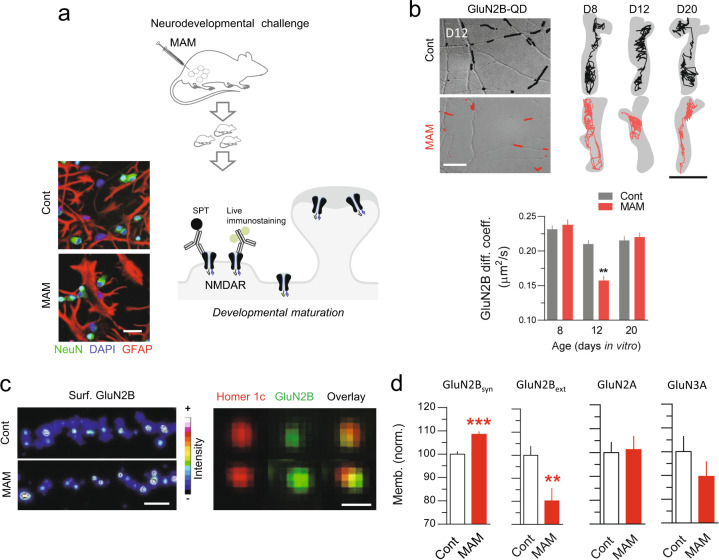
NMDAR surface dynamics and synaptic plasticity are developmentally impaired in the MAM model. **a** Schematic description of the experimental workflow. Cultured hippocampal networks were made from rat pups. Examples of immuncytochemical staining of neurons (NeuN, green), nucleus (DAPI, blue), and glial cells (GFAP, red) in cultured network at 10 days in vitro from control or MAM-exposed pups. **b** Representative trajectories (50 ms acquisition) of surface GluN2B-NMDAR-QD complexes on neurons from control or MAM-exposed pups at three developmental stages: days in vitro 6–10 (D8), days in vitro 10–15 (D12), days in vitro 15–22 (D20). Scale bars = 5 µm/300 nm (left/right). Right, Comparison of GluN2B-NMDAR diffusion coefficient control or MAM-exposed conditions (D8 cont, *n* = 1916 trajectories; D8 MAM, *n* = 1220; D12 cont, *n* = 2055; D12 MAM, *n* = 946; D20 cont, *n* = 1288; D20 MAM, *n* = 1317; **p < 0.01, Mann–Whitney test). Data are represented as mean ± sem. **c** Surface immunostaining of GluN2B-NMDAR onto neurons from control or MAM-exposed pups (synapses were detected with Homer1c-GFP). Scale bars = 5/1 µm (left/right). **d** Comparison of the GluN2B, GluN2A, GluN3A-NMDAR cluster areas between conditions (GluN2B synaptic cont, *n* = 2144 clusters; GluN2B synaptic MAM, *n* = 2468, ***p < 0.001, Student’s *t*-test; GluN2B extrasynaptic cont, *n* = 1733; GluN2B extrasynaptic MAM, *n* = 1421, **p < 0.01, Student’s *t*-test; GluN2A cont, *n* = 56; GluN2A MAM, *n* = 41; GluN3A cont, *n* = 59; GluN3A MAM, *n* = 48).

A wealth of past and recent studies supports the hypothesis that SCZSD originates early in brain development, during which synaptic and neuronal network maturation are impaired [32, 33, 49]. Several rodent developmental models of SCZSD have been established and used to track down some of the cellular and molecular disturbances that may be of great translational potential. Among these, the administration of mitotoxin MAM in pregnant female rats produces synaptic, pharmacological, anatomical, and behavioral impairments in offspring once they reach adulthood [35]. We thus used this developmental SCZSD model to test whether NMDAR surface dynamics would be impaired over development. As expected, the weight of MAM-exposed newborn pups, the total number of cells and the number of newborn cells in the hippocampus were significantly reduced after birth (P2) in MAM-exposed (Supplementary Fig. 3). In cultured hippocampal cells from MAM-exposed pups, both glial cells (labeled with GFAP) and neurons (labeled with NeuN) were present and well-developed (Fig. 4a). We then imaged the NMDAR surface dynamics at the surface of developing hippocampal neurons. Based on the in vitro maturation profile of glutamatergic synapses (Supplementary Fig. 4), we focused at three developmental stages: before (div 6–8, D8), during (div 10–14, D12) and after (div 18-22, D20) synaptogenesis. The density of dendritic spines in mature hippocampal neurons was undistinguishable between control and MAM-exposed neurons (Supplementary Fig. 3c). Yet, the NMDAR surface dynamics was strongly impaired at the peak of synaptogenesis, i.e., in the D12 time window, with no significant change at D8 or D20 (Fig. 4b). At D12, the intensity of synaptic NMDAR clusters was significantly increased, consistent with the depletion of the extrasynaptic pool of GluN2B receptors (Fig. 4c, d). Noteworthy, this change appears to be specific to GluN2B-NMDAR, which is expressed early in development, since there was no change in GluN2A- or GluN3A-NMDAR content (Fig. 4c, d).

**Fig. 5:**
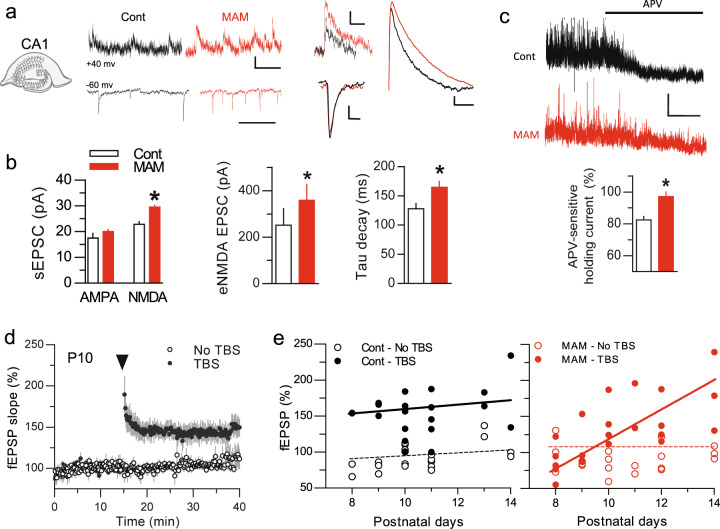
NMDAR-mediated transmission and NMDAR-dependent LTP is altered in MAM pups. **a** Spontaneous AMPAR and NMDAR-mediated EPSCs recorded in whole-cell configuration at −70 and +40 mV, respectively, in the CA1 of hippocampal slices from control and MAM-exposed animals. Insets show superimposed examples of spontaneous AMPAR and NMDAR-mediated responses as well as evoked NMDAR-mediated currents. **b** Spontaneous (*n*_(cont/MAM)_ = 14/20; *p* < 0.05) as well as evoked (*n*_(cont/MAM)_ = 16/16; *p* < 0.05) NMDAR-mediated currents are significantly larger in MAM-exposed animals. Also, the Tau decay was increased by ~25% (*n*_(cont/MAM)_ = 8/16; *p* < 0.05), which is consistent with an increased synaptic expression of GluN2B. **c** Tonic NMDA currents were recorded as the subtracted holding current at a depolarized potential of +40 mV before and after the addition of APV. These tonic NMDA currents were significantly decreased in MAM-exposed animals (*n*_(cont/MAM)_ = 10/10; *p* < 0.05), as revealed by a much smaller decrease in holding current with APV. **d** Field excitatory postsynaptic potential (fEPSP) recorded in CA1 pyramidal cell layer after stimulation of hippocampal Schaffer collaterals. Active input displays a significant postsynaptic potentiation (LTP) following high-frequency stimulation at P10 (control), not visible in non-stimulated input (*n* = 6; *p* < 0.05). **e** When comparing the response to high-frequency stimulation in control and MAM-exposed animals there was a clear deficit in the magnitude of the LTP in MAM-exposed rats between P8 and P12. This deficit was transient and disappeared with age such that the LTP recovered in older animals.

We next recorded the CA3-CA1 synaptic transmission in acute brain slices from control and MAM-exposed rats at different postnatal days. At postnatal day 10 (P10), which is a time of peak synaptogenesis, the amplitude of AMPAR-mediated spontaneous EPSCs was indistinguishable between control and MAM-exposed rats (Fig. 5a). In contrast, the amplitude of NMDAR-mediated spontaneous EPSCs was significantly increased in MAM-exposed rats (Fig. 5a, b), consistent with the above in vitro data. We further confirmed this amplitude increase in evoked NMDAR-mediated EPSCs (Fig. 5b). In addition, the kinetics of evoked NMDAR-mediated EPSC was altered in MAM-exposed rats: the decay time was increased by ~25% (Fig. 5b), consistent with the increase contribution of GluN2B-NMDAR in synaptic clusters. Finally, we measured the tonic current of NMDAR-mediated in CA1 pyramidal neurons since we observed a decrease in the content of extrasynaptic NMDAR in cultured hippocampal pyramidal neurons (Fig. 4f). For this, we recorded the APV-sensitive holding current in control and MAM-exposed rats (Fig. 5c). In control condition, a tonic NMDAR current was clearly detectable (Fig. 5c), as previously described [39]. However, this current was absent in MAM-exposed rats (Fig. 5c). Altogether, these in vitro and ex vivo data show that MAM-exposed rats exhibit a deficit in GluN2B-NMDAR surface dynamics and an accumulation at synapses during their peak of maturation. To test whether these major alterations alter the functional properties of the maturing glutamate synapse, we induced NMDAR-dependent synaptic long-term potentiation (LTP) using a high-frequency stimulation protocol (Fig. 5d) in both conditions and at various developmental stages. Although LTP could be induced in control rats during the second postnatal week (from P8 to P14), a deficit in LTP was clearly observed between P8 and P12 in MAM-exposed rats (Fig. 5e). Collectively, these data demonstrate that MAM exposure during prenatal life corrupt NMDAR surface dynamics, synaptic content, and NMDAR-dependent LTP during the period of synaptic maturation.

### Tuning GluN2B-NMDAR NMDAR surface dynamics prevents behavioral deficit in adult MAM rats

**Fig. 6:**
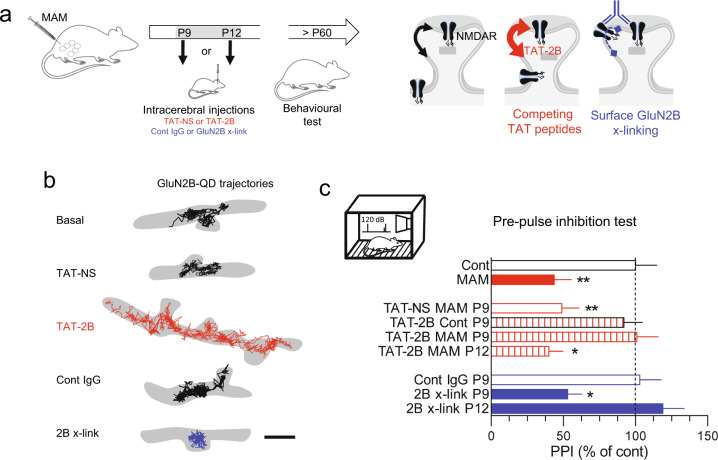
Tuning GluN2B-NMDAR NMDAR surface dynamics alters sensorimotor gating in adults. **a** Schematic description of the experimental workflow: control of MAM-exposed pups received intracerebral injections during the second postnatal week of NMDAR surface diffusion modulators. **b** Representative trajectories (50 ms acquisition) of surface GluN2B-NMDAR on hippocampal neurons exposed to TAT-non sense (TAT-NS, 10 µM), TAT-2B (10 µM), control IgG (10 µg/ml), or IgG direct against extracellular epitope of the GluN2B-NMDAR (10 µg/ml). Scale bars = 500 nm (left/right). **c** The startle response, i.e., pre-pulse inhibition (PPI), was measured at P60 and compared between conditions (*n* = 10–16 rats per group; *P < 0.05, **P < 0.01, ANOVA 1 followed by Newman–Keuls multiple comparisons test).

The fact that GluN2B-NMDAR surface dynamics is specifically downregulated during the postnatal development of MAM-exposed rats prompted us to test whether this early life event was associated to the emergence of the adult behavioral deficits repertoire. Our experimental strategy consisted in rescuing the GluN2B-NMDAR surface trafficking in MAM-exposed rats by upregulating it during the second postnatal week (Fig. 6a). For this, we used a competing ligand, composed of the last 15 amino acids of the GluN2B C-terminus and coupled to a TAT sequence in order to penetrate within cells, that prevent the interaction between GluN2B-NMDAR and PDZ scaffold proteins in synapses and consequently increase GluN2B-NMDAR surface dynamics (Fig. 6a, b) [38, 50]. The TAT-2B ligand, or its non-sense control version (i.e., TAT-NS), was injected in the dorsal hippocampus of control or MAM-exposed rats at (1) P9 in order to favor NMDAR dynamics during the sensitive altered period, or (2) at the end of the sensitive period (P12) (Fig. 6a). At adult stage (P60-65), the synaptic content of NMDAR subunits, measured by western blot in synaptosomes, was not significantly different between control, MAM-exposed, and TAT-2B-injected (P9) rats (Supplementary Fig. 5). We then used the gold-standard PPI test in adult rats, a behavioral assay consistently affected in MAM-exposed rats [35, 51]. We first confirmed that MAM-exposed rats poorly responded to PPI (Fig. 6c). Remarkably, the PPI deficit was abrogated in MAM-exposed rats when the TAT-2B ligand was injected at P9; the TAT-NS in MAM-exposed rats or TAT-2B in control rats had no effect (Fig. 6c). When the TAT-2B ligand was injected later, at P12, MAM-exposed rats still exhibit a poor response to PPI (Fig. 6c). Note that the reactivity to the startle alone was not difference between groups (Control: 48 ± 3 ms; MAM: 43 ± 3; MAM TAT-NS: 37 ± 10; MAM TAT-2B (P9): 43 ± 6; MAM TAT-2B (P12): 45 ± 3; ANOVA 1, *F* = 0.735, *P* = 0.57). Thus, the artificial increase in GluN2B-NMDAR surface dynamics during the second postnatal week is sufficient to prevent the PPI deficit observed in adult MAM rats.

These results suggest thus the impairment in NMDAR surface diffusion during the second postnatal week produce behavioral deficits at adulthood. To directly test this possibility, we used a cross-link (x-link) strategy [20, 52, 53]. Schematically, surface NMDAR are x-linked by an antibody directed against an extracellular epitope of the GluN2B subunit, which resulted in drastic reduction of the receptor surface dynamics (Fig. 6a, b). Anti-GluN2B or control antibodies were injected in the dorsal hippocampus at P9. At adult stage (P60-65), the synaptic content of NMDAR subunits, measured by western blot in synaptosomes, was not significantly different between control and GluN2B-x-link injected rats (Fig. 6b). When the surface dynamics of GluN2B-NMDAR was impaired from P9, the PPI response at adulthood was significantly impaired (Fig. 6c). However, when the injection was performed at P12, the PPI response was unaltered (Fig. 6c). Note that the reactivity to the startle alone was not difference between groups (Cont IgG (P9): 58 ± 8 ms; X-link2B (P9): 49 ± 4; X-link2B (P12): 57 ± 4; ANOVA 1, *F* = 0.872, *P* = 0.43). Collectively, these data unveil that during the second postnatal week, the NMDAR surface dynamics, and consequently its synaptic content, is essential for the proper establishment of behavioral responses at adulthood.

## Discussion

Dysfunctions of the glutamatergic synaptic transmission and neuronal network activities have been hallmarks of psychosis models. Here, we investigated and compared the trafficking of NMDAR in gold-standard models of psychosis using a combination of single-molecule tracking, electrophysiological, immunohistochemical, biochemical, and behavioral approaches. We unveil that NMDAR surface dynamics is consistently impaired in genetic and developmental models of psychosis. Furthermore, CSF from patients suffering from SCZSD, but not from other neurological or psychiatric conditions, impair NMDAR surface trafficking and synaptic content in hippocampal cell network. These data shed thus new light on how the glutamatergic NMDAR signaling is corrupted in models of psychosis, further strengthening the glutamatergic hypothesis of psychotic disorders.

In the brain, neurons are surrounded with the interstitial fluid that has a chemical composition very close to the CSF. Identifying the putative molecular change in the CSF of patients suffering from SCZSD or other psychotic disorders has been an active area of investigation. There is a consensus that the CSF from patients with SCZSD is different from the one of healthy individuals [54]. For instance, the neopterin CSF concentration, which relate to the inflammatory status, is upregulated in SCZSD patients when compared to healthy individuals [55]. Kynurenine metabolites (e.g., kynurenic and quinolinic acids) and cytokines (e.g., IL-6, TNF-a, IL-8) also appear to be altered in SCZSD CSF [37, 56–58]. Besides their role in inflammation and cellular signaling, most of these molecules can also modulate the glutamatergic synaptic transmission, including the NMDAR-mediated one (e.g., kynurenic acid is a NMDAR antagonist) [33]. The CSF content of neuroplasticity-associated proteins, such as neurexin, amyloid precursor protein, glial cell-derived neurotrophic factor, hepatocyte growth factor, and S100 calcium-binding protein B, is also altered in patients with SCZSD [59, 60]. Furthermore, the brain content of the NMDAR agonist and co-agonists, i.e., glutamate, glycine, and D-serine, appear to be decreased in patients with SCZSD, although there is no consensus yet between studies [61, 62]. Thus, the NMDAR signaling is putatively altered by a myriad of molecules whose expression is corrupted in the brain of patients with SCZSD. Which of these factors could increase the membrane dynamics of NMDAR reported in this study? Although there is currently no straightforward answer to this question, one can speculate that the receptor agonist, co-agonist, and cytokines are, among others, putative contributors to the cellular phenotype. Indeed, NMDA, D-serine, and glycine have been shown to significantly decrease the surface dynamics of NMDAR onto hippocampal neurons [40, 63, 64]. The reported low levels of NMDAR agonist and co-agonists in the CSF of patients with SCZSD would thus upregulate the receptor surface dynamics. In the same vein, the CSF content of cytokines (e.g., IL-1, IL-6) is increased in the CSF of patients with SCZSD, and cytokines are potent in upregulating NMDAR surface dynamics [65]. Future investigations are surely needed to unveil the mechanism underpinning the corrupted NMDAR surface dynamics in SCZSD patients. Whether a single molecule or a combination of different molecules induces NMDAR dysfunction is an essential, yet complex, challenge to tackle. With the prism of the mild encephalitis hypothesis in SCZSD [66], the variety of molecular stigmata identified in the CSF of patients with SCZSD (e.g., intrathecal humoral immune responses, increased CSF cell counts, oligoclonal IgG, blood–CSF barrier dysfunctions, autoantibodies, elevated neopterin level) suggest either the presence of a mosaic of different subgroups of patients with different monofactorial molecular process, or a single multifactorial molecular process for most patients, that lead to an unbalanced neurotransmitter system communication. Our study indicates that the NMDAR surface trafficking was consistently altered in patients with SCZSD, suggesting a convergence point between all the putative molecular dysfunction combinations.

Animal models have been critical to investigate the basis of neuropsychiatric disorders since they permit a molecular and cellular dissection of the pathophysiology. Among the gold-standard models that emerged from these translational attempts, the genetic manipulations based on human studies (e.g., DISC1) as well as developmental models in which the establishment of synapses and networks is corrupted during a critical neonatal period [32–36] have received a lot attention. Here, we used two of these models to directly question whether the NMDAR surface dynamics is impaired in these conditions. First, we manipulated the expression of DISC1 whose disruption by a chromosomal translocation or other mutations has been proposed to predispose the person to a number of neuropsychiatric disorders including SCZSD [42, 43], although this remains a matter of debate [67]. Since DISC1 is found in the glutamatergic synapse [43] and durably alters the NMDAR-mediated transmission and synaptic plasticity [44, 45, 68], we measured the surface dynamics of NMDAR in hippocampal neurons in which DISC1 expression was downregulated, and report a consistent deficit in NMDAR surface dynamics. This suggests that DISC1 regulates the organization of the postsynaptic density that is essential for the proper stabilization and surface anchoring of NMDAR. In rodent models, mutation or downregulation of DISC1, mainly during the first postnatal weeks, impair neuronal network establishment, sensory integration, and lead to behavioral deficit at adulthood [46, 69, 70]. It is thus possible that DISC1 deficit during the critical phase of postnatal synaptic maturation and neuronal network development profoundly and durably corrupt the brain through dysfunctional NMDAR signaling. We thus used a classical developmental model of SCZSD, based on the injection of MAM in pregnant rat female [35], to test whether NMDAR surface dynamics is altered during the critical period of synaptic and network maturation. In MAM-exposed pups, the NMDAR expression is altered early in development [71]. Here, we further unveil that during the second postnatal week (corresponding here to 10–14 days in vitro for cultured hippocampal networks) in MAM-exposed pups the NMDAR surface dynamics, NMDAR-mediated basal transmission, and NMDAR-dependent LTP is corrupted. Intriguingly, these deficits appear to be reversible as they recover basal value later on. Artificial manipulations of the NMDAR surface dynamics during the second postnatal week could either mimic or prevent the behavioral deficit in MAM-exposed rats, suggesting that the altered receptor surface dynamics contribute to the adult phenotype. Consistently, it has recently been shown in another SCZSD model of developmental inflammation that the NMDAR surface dynamics is severely impaired early in development, and the adult behavioral deficits could be prevented by regulating NMDAR surface dynamics during the second postnatal week [65]. Collectively, past and present studies fuel a model in which the proper maturation of synapses and networks, during the first postnatal weeks in rodents, is corrupted in SCZSD possibly through a pathological regulation of NMDAR surface dynamics and synaptic organization. This prompts the development of new and innovative strategies to regulate the NMDAR signaling in SCZSD, not with ionotropic regulators that unfortunately clinically failed over the past decades, but rather through receptor membrane stabilizer and trafficking regulator [23]. In addition, developing new approaches to estimate the overall NMDAR dynamics in patients with SCZSD may provide an additional clinical measure and may eventually constitute a new biomarker of the disorder.

## Supplementary information

SF 1

SF 2

SF 3

SF 4

SF 5
