# Supplementary material for: Alteration of NMDA receptor trafficking as a cellular hallmark of psychosis
Source: Transl Psychiatry. 2021 Aug 30;11:444. doi: 10.1038/s41398-021-01549-7 (PMC8405679; doi:10.1038/s41398-021-01549-7)
Supplement: Supplementary file 1 — SF 1 [file 41398_2021_1549_MOESM1_ESM.pdf]

Suppl. Figure 1

Espana, Seth et al.

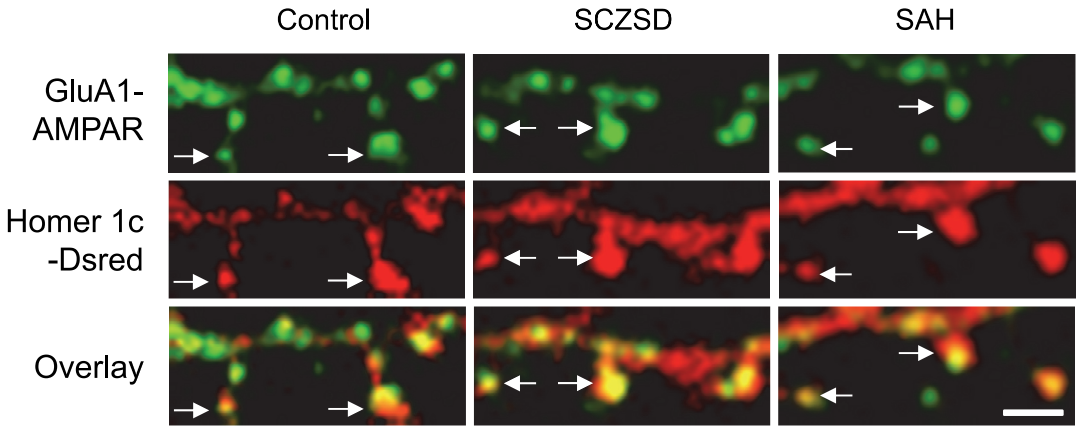

Suppl. Fig. 1. Surface immunostaining of GluA1-AMPAR-SEP (green) onto neurons transfected with Homer1c-DsRed (red) and exposed to SCZSD or SAH CSF. Arrows indicate postsynaptic areas. Scale bar = 1 µm.
