# Supplementary material for: Alteration of NMDA receptor trafficking as a cellular hallmark of psychosis
Source: Transl Psychiatry. 2021 Aug 30;11:444. doi: 10.1038/s41398-021-01549-7 (PMC8405679; doi:10.1038/s41398-021-01549-7)
Supplement: Supplementary file 2 — SF 2 [file 41398_2021_1549_MOESM2_ESM.pdf]

# Suppl. Figure 2

Espana, Seth et al.

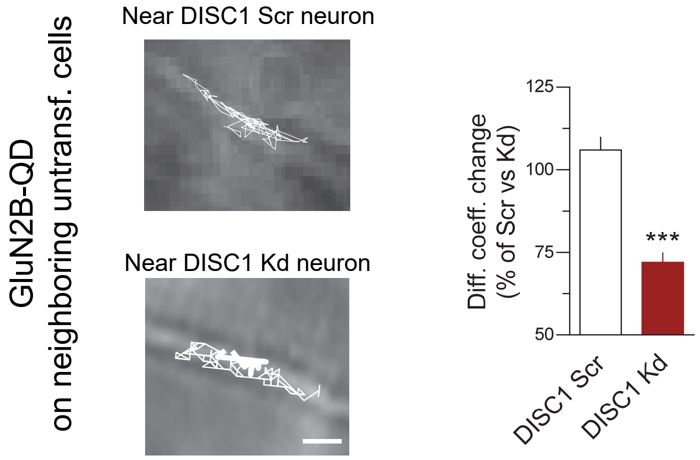


**Suppl. Fig. 2.** DISC1 downregulation does not alter GluN2B-NMDAR surface dynamics in untransfected neighboring neurons. Left, Representative trajectories (50ms acquisition) of surface GluN2B-NMDAR-QD complexes on untransfected neighboring neurons near a transfected neuron with either a scramble siRNA (DISC1 Scr) or DISC1 siRNA (DISC1 Kd). Scale bar = 500 nm. *Right*, Comparison of the diffusion coefficient in untransfected neighboring neurons near a transfected neuron with either a scramble siRNA (DISC1 Scr) or DISC1 siRNA (DISC1 Kd) (n = 4 coverslips per conditions; ***p<0.001, Mann-Whitney test).
