# Supplementary material for: Alteration of NMDA receptor trafficking as a cellular hallmark of psychosis
Source: Transl Psychiatry. 2021 Aug 30;11:444. doi: 10.1038/s41398-021-01549-7 (PMC8405679; doi:10.1038/s41398-021-01549-7)
Supplement: Supplementary file 3 — SF 3 [file 41398_2021_1549_MOESM3_ESM.pdf]

Suppl. Figure 3

Espana, Seth et al.

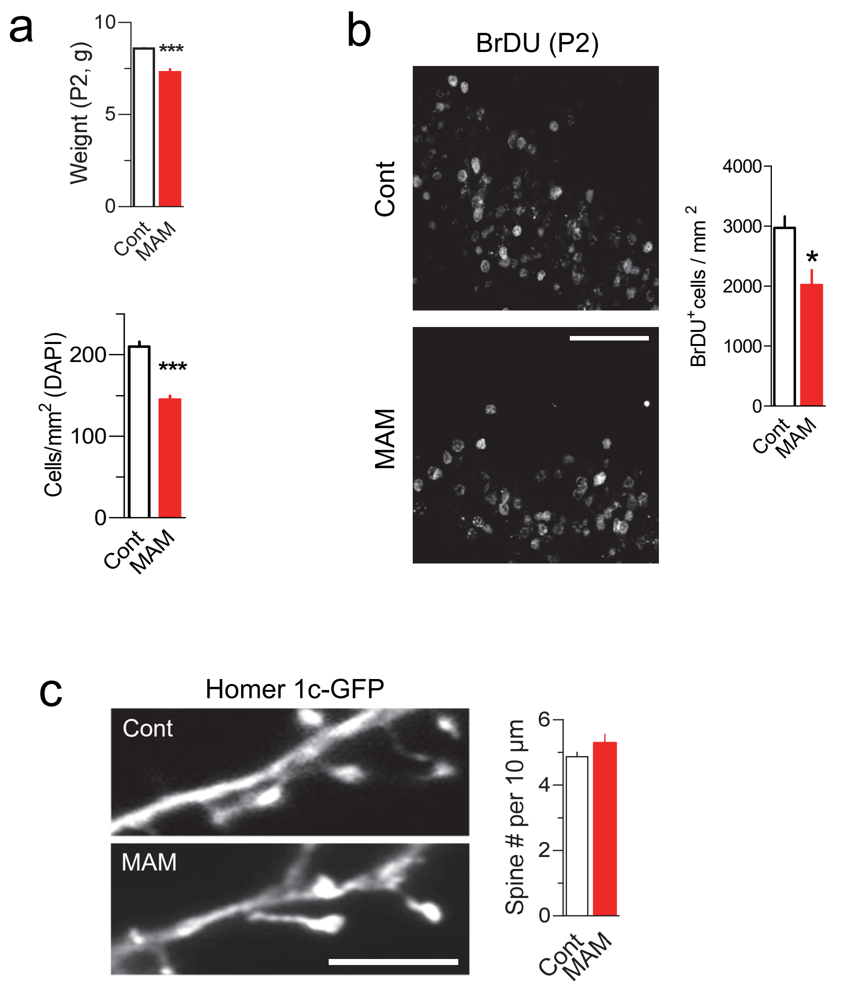

Suppl. Fig. 3. Characteristics of the brain tissue and cells in MAM-exposed pups. **a** At P2, the body weight of the MAM-exposed pups was reduced when compared to control littermates (n = 94 and 88 control and MAM-exposed pups, respectively; *** p<0.001, Student-t test). The cell density in the CA1 area of the hippocampus, estimated with DAPI staining, was also reduced in MAM-exposed pups when compared to control pups (n = 383 and 361 fields in control and MAM-exposed pups, respectively; *** p<0.001, Student-t test). **b** The density of newborn cells in the CA1 area of the hippocampus, estimated with BrDU staining, was reduced in MAM-exposed pups when compared to control pups (n = 6 animals in control and MAM-exposed pups, respectively; *p<0.05, Student-t test). Scale bar = 2 mm. **c** At 10 days *in vitro,* the linear density of dendritic spines, defined as protusions containing Homer1c-GFP clusters, was similar in MAM-exposed pups when compared to control pups (n = 42 and 27 fields in control and MAM-exposed pups, respectively; p>0.05, Student-t test). Scale bar = 1.5 µm.
