# Supplementary material for: Alteration of NMDA receptor trafficking as a cellular hallmark of psychosis
Source: Transl Psychiatry. 2021 Aug 30;11:444. doi: 10.1038/s41398-021-01549-7 (PMC8405679; doi:10.1038/s41398-021-01549-7)
Supplement: Supplementary file 4 — SF 4 [file 41398_2021_1549_MOESM4_ESM.pdf]

Suppl. Figure 4

Espana, Seth et al.

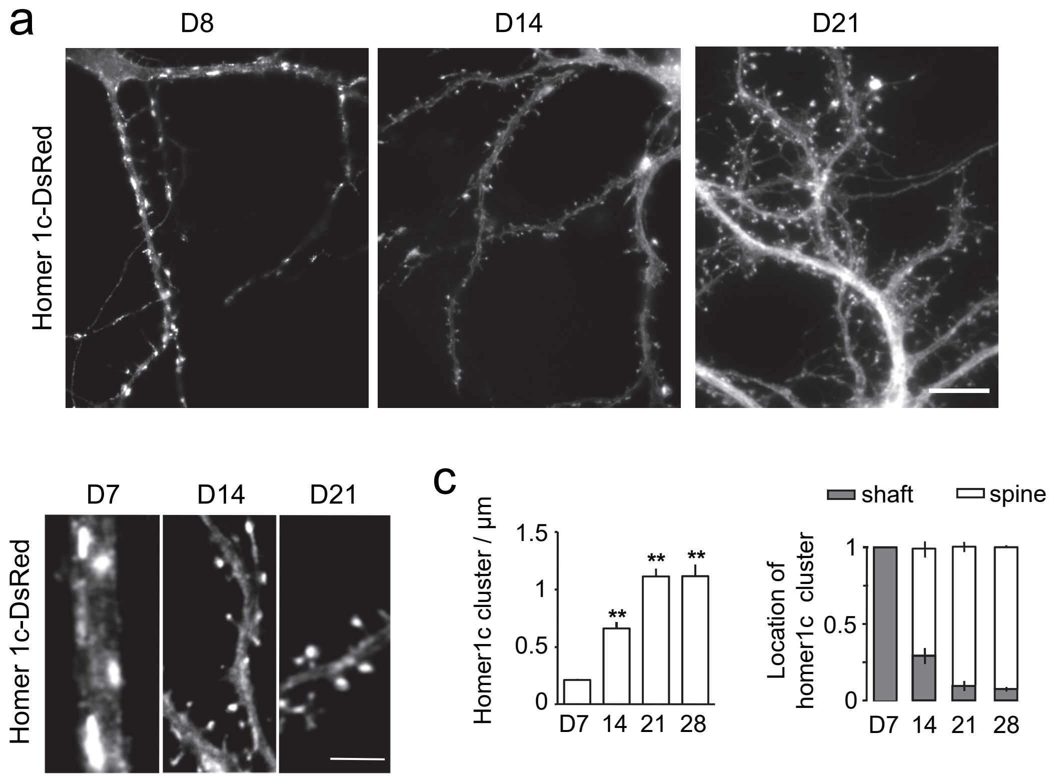

Suppl. Fig. 4. Developmental profile of the synaptogenesis in cultured hippocampal neurons at different days *in vitro* (D). **a** Fluorescence imaging of Homer 1c-DsRed at D8, 14, and 21. Note the lack of dendritic spine at D8 and their increasing appearance from D14. Scale bar = 10 µm. **b** Enlarged images from a. Scale bar = 2 µm. **c** Quantification of the linear density of Homer 1c cluster (/um of dendrite) at D7, 14, 21, and 28 (n = 20 fields per developmental stage; **p<0.01, ANOVA 1 followed by Newman-Keuls multiple comparisons test). The localization of the Homer 1c cluster on dendritic shaft or spine was also quantified.
