# Supplementary material for: Alteration of NMDA receptor trafficking as a cellular hallmark of psychosis
Source: Transl Psychiatry. 2021 Aug 30;11:444. doi: 10.1038/s41398-021-01549-7 (PMC8405679; doi:10.1038/s41398-021-01549-7)
Supplement: Supplementary file 5 — SF 5 [file 41398_2021_1549_MOESM5_ESM.pdf]

Suppl. Figure 5

Espana, Seth et al.

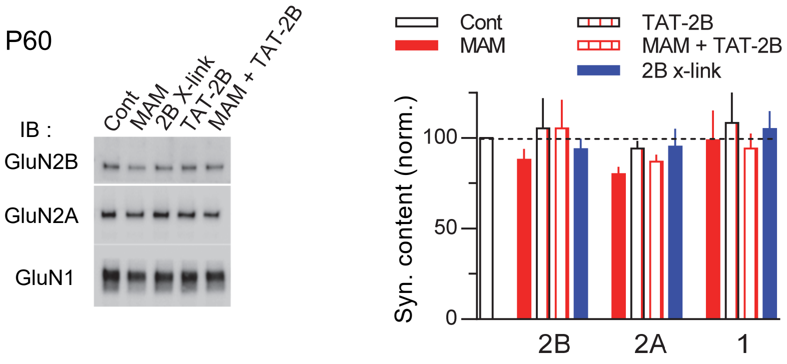

Suppl. Fig. 5. At postnatal day 60 (P60), synaptosomes were prepared from hippocampal tissue and GluN1, 2A, 2B subunit content was measured by western blot (n = 3-6 rats per group). There was no significant difference between conditions (p>0.05).
